# Modified ulnar lengthening for correction of the Masada type 2 forearm deformity in hereditary multiple exostosis

**DOI:** 10.1038/s41598-023-37532-z

**Published:** 2023-06-29

**Authors:** Shu Cao, Jian-Fa Zeng, Sheng Xiao, Zhong-Gen Dong, Zi-Li Xu, Hong Liu, Xin Li, Ke Fang, Jie Wen, Ming Zeng, Zhong-Wen Tang, Bo Li, Hao-Li Gong, Fan-Ling Li

**Affiliations:** 1 Department of Orthopedics, Hunan Provincial People’s Hospital, The First Affiliated Hospital of Hunan Normal University, Changsha, People’s Republic of China; 2 Department of Orthopedics, The Second Xiangya Hospital, Central South University, No. 139 Renmin Road, Changsha, 410011 Hunan People’s Republic of China

**Keywords:** Diseases, Medical research

## Abstract

Few articles have reported on the treatment of Masada type 2 forearm deformities in hereditary multiple exostosis, possibly because of the high redislocation rate and other complications. This study precisely declares the use of modified ulnar lengthening by an Ilizarov external fixation with tumour excision for the treatment of Masada type 2 forearm deformities. 20 children with Masada type 2 forearm deformities were admitted for surgical treatment at our hospital from February 2014 to February 2021. There were 13 girls and 7 boys, ranging in age from 3.5 to 15 years (mean: 9 years) at the time of operation. We removed the prominent osteochondromas of the distal ulna and the proximal radius, positioned a classic Ilizarov external fixator on the forearm and then performed ulnar transverse one-third proximal diaphyseal subperiosteal osteotomy. We adopted modified ulnar lengthening postoperatively. The effects of surgical correction of deformity and functional improvement of the limb were assessed via regular follow-up and X-ray. The patients were followed up for 36 months, and the ulna was lengthened 26.99 mm on average; all radial heads remained relocated. The radiographic evaluations, including relative ulnar shortening, radial articular angle, and carpal slip, were improved. The functions of the elbow and forearm were all improved after surgery. Modified ulnar lengthening by an Ilizarov external fixation with tumour excision for the treatment of Masada type 2 forearm deformities in hereditary multiple exostoses has been proven to be an effective and reliable technique in the early stage.

## Introduction

Hereditary multiple exostoses (HME), called diaphyseal aclasis, is a disorder of endochondral bone growth at the metaphysis of a bone in a patient with a family history^1–3^. HME is an autosomal dominant skeletal disorder involving two virulence genes, EXT1 and EXT2, located at 8q24.11 and 11p11.2, respectively^4–6^. The clinical manifestations of HME include a bone mass, abnormal appearance and limb-function impairment^7,8^. The exostoses often invade the metaphysis and hinder growth of the epiphysis of the long bone, leading to defective metaphyseal remodelling and asymmetrical retardation of longitudinal bone growth. This results in progressive bone shorting and deformity in immature patients, especially in the distal epiphysis of the ulnar; thus, 30–70% of patients have forearm deformity^1,9–11^. The main deformities of the forearm on radiography are a relative shortening of the ulna, a secondary bowing of either or both forearm bones, increased ulnar tilt of the distal radial epiphysis, ulnar deviation of the hand, progressive ulnar translocation of the carpus, and dislocation of the radial head in severe cases^7^. All of these deformities can lead to clear pseudo-varus deformity of the elbow and dysfunction of the forearm. Radial head dislocation may result in poor elbow function, pain, and limb deformities^7,9^. Classical evaluation of the forearm deformity was divided into 3 types, according to the system of Masada et al.^12^ patients with type 1 and type 2 deformities were more common.

Several procedures have been used to correct forearm deformities, including simple excision of osteochondromas, acute or gradual ulnar lengthening, hemiepiphysiodesis, radial head resection, corrective radial osteotomy, creation of a one-bone forearm and the Sauvé–Kapandji procedure^1,9,13–15^. However, there is no common consensus regarding the most appropriate operative intervention for the treatment of radial head dislocation due to HME in paediatric patients. Some good results have been achieved in patients with no radial head dislocation after ulnar lengthening^1,12,13,16^. However, for paediatric patients with radial head dislocation (Masada type 2), controversy remains concerning the difficulty to achieve a reduced humeroradial joint, abnormalities in the distal radioulnar joint and the high recurrence dislocation of the radial head during follow-up^17^_._

In this present study of paediatric patients with radial head dislocation in association with hereditary multiple exostoses, we managed with modified ulnar lengthening and then evaluated the outcome in terms of radial head dislocation, forearm function and radiological results during follow-up. The purpose of this study was to evaluate the effect of the modified ulnar distraction by Ilizarov external fixation and deformity correction.

## Results

The results are listed in Tables 1, 2 and 3. Among the patients, Cases 2 and 3 were siblings, and their mother also suffers from hereditary multiple exostosis. All resected tumours were pathologically confirmed as exostoses, and none were malignant. At the latest follow-up, all radial heads remained located. The mean follow-up time was 36 months (range: 24–60 months). The mean distraction distance was 26.99 ± 12.96 mm (range: 10.43–66.61 mm). The average distraction period was 38.35 ± 21.24 days (range: 15–105 d). The period of fixator treatment averaged 182 ± 59.15 days (range: 90–354 d). The mean DI was 14.27 ± 2.70 days/cm (range: 8.25–19.79 d/cm). The EFI averaged 73.55 ± 21.98 days/cm (range: 44.94–124.54 mm/d) (Table 1).

**Table 1:** Demographics of patients.

Patient	Age (years)	Gender	Side	Masada type	Length gain (mm)	Duration of distraction (days)	Duration of external fixation (days)	Distraction index (days/cm)	External fixation index (days/cm)
Case 1	11.6	F	R	2b	31.68	35	223	11.05	70.39
Case 2	6	M	R	2b	38.78	32	218	8.25	56.21
Case 3	11	F	L	2b	39.38	62	193	15.74	49
Case 4	12	F	R	2b	35.26	44	213	12.48	60.41
Case 5	9.1	M	R	2a	41.61	72	187	17.3	44.94
Case 6	5.3	F	L	2b	66.61	105	354	15.76	53.15
Case 7	7.4	M	L	2a	14.55	26	114	17.87	78.35
Case 8	10.4	F	R	2a	22.58	28	267	12.4	118.25
Case 9	11	F	R	2b	26.75	34	193	12.71	72.15
Case 10	9.1	M	L	2a	33.63	60	187	17.84	55.61
Case 11	3.5	F	R	2b	10.43	15	90	14.38	86.29
Case 12	15	F	L	2b	20.77	27	160	13	77.03
Case 13	11.5	F	R	2a	16.38	24	204	14.65	124.54
Case 14	11.2	F	R	2a	19.21	25	180	13.01	93.07
Case 15	9.1	F	L	2b	19.38	26	165	13.42	85.14
Case 16	9.1	F	R	2b	21.91	29	165	13.24	75.31
Case 17	11	F	R	2b	24.22	34	150	14.04	61.93
Case 18	5.8	M	L	2a	14.15	28	136	19.79	96.11
Case 19	6.3	M	L	2a	20.31	25	114	12.31	56.13
Case 20	5	M	R	2a	22.30	36	127	16.14	56.95

**Table 2:** Radiologic results of patients.

Patient	Radial articular angle (°)		Carpal slip (%)		Ulnar shortening (mm)	
	Preoperative	At last follow-up	Preoperative	At last follow-up	Preoperative	At last follow-up
Case 1	37	18	68.22	35.17	− 11.06	0
Case 2	28	11	43.21	30.21	− 13.9	0
Case 3	47	28	64.65	38.33	− 19.59	0
Case 4	39	34	60.33	50.00	− 13.85	0
Case 5	31	27	77.34	50.00	− 9.17	0
Case 6	28	30	70.18	76.61	− 12.46	− 2.29
Case 7	39	37	100.00	42.10	− 8.21	0
Case 8	44	28	62.49	49.37	− 18.32	− 11.34
Case 9	47	45	64.65	36.48	− 7.41	0
Case 10	34	30	69.28	50.00	− 6.51	0
Case 11	33	28	100	63.64	− 10.61	− 5.50
Case 12	46	32	48.57	57.63	− 6.00	0
Case 13	36	26	46.85	30.63	− 4.62	0
Case 14	37	39	51.81	52.55	− 7.02	0
Case 15	35	42	52.86	24.58	− 9.66	− 4.62
Case 16	35	47	52.10	62.10	− 7.85	0
Case 17	37	29	58.54	27.13	− 9.33	0
Case 18	33	20	64.38	57.57	− 12.23	− 4.38
Case 19	31	29	67.00	50.68	− 13.92	0
Case 20	35	27	100.00	100.00	− 13.58	0

**Table 3:** Range of motion of the forearm.

Patient	Supination-pronation (°)		Flexion–extension of the elbow (°)	
	Preoperative	At last follow-up	Preoperative	At last follow-up
Case 1	85–65	90–70	110-Full	120-Full
Case 2	80–80	80–80	125-Full	130-Full
Case 3	75–75	80–80	110-Full	120-Full
Case 4	80–65	90–80	120-Full	120-Full
Case 5	70–60	80–75	100-Full	120-Full
Case 6	60–60	80–80	90-Full	120-Full
Case 7	70–55	85–75	110-Full	120-Full
Case 8	70–60	70–70	130-Full	130-Full
Case 9	80–75	80–80	120-Full	130-Full
Case 10	70–70	85–80	110-Full	120-Full
Case 11	80–40	80–80	90-Full	110-Full
Case 12	90–45	90–70	100-Full	110-Full
Case 13	60–50	80–70	110-Full	120-Full
Case 14	65–55	80–60	100-Full	110-Full
Case 15	60–55	85–70	120-Full	125-Full
Case 16	65–60	80–70	105-Full	120-Full
Case 17	70–70	80–70	100-Full	115-Full
Case 18	65–75	80–80	115-Full	115-Full
Case 19	75–50	85–70	90-Full	115-Full
Case 20	60–50	80–70	110-Full	120-Full

### Radiologic assessment

The radiologic parameters were improved. RAA improved from 36.60° to 30.35°, and CS improved from 66.12 to 49.24%. The shortening of the ulna was reduced from 10.77 to 1.41 mm. These improvements were highly statistically significant (Tables 2 and 4).

**Table 4:** Statistical comparison of patients’ data preoperation and the latest follow-up.

	RAA (°)	CS (%)	US (°)	Supination (°)	Pronation (°)	Elbow flexion (°)
	(Mean ± SD)	(Mean ± SD)	(Mean ± SD)	(Mean ± SD)	(Mean ± SD)	(Mean ± SD)
preoperative	36.60 ± 5.73	66.12 ± 17.00	− 10.77 ± 3.99	72 ± 9	61 ± 11	108 ± 12
Last follow-up	30.35 ± 8.76	49.24 ± 18.05	− 1.41 ± 2.95	82 ± 5	74 ± 1	119 ± 6
t	3.29	4.36	− 10.77	− 5.92	− 6.02	− 6.93
*P* value	0.004	0.000	0.000	0.000	0.000	0.000

RAA, radial articular angle; CS, carpal slip; US, Ulnar shortening.

*Student’s t test;* P* ≤ 0.05, statistically significant at.

### Functional assessment

At the time of final follow-up, the mean forearm supination was 82.00°, and the mean forearm pronation was 74°. The mean elbow flexion was 119.00°, and the motion of elbow extension was not a preoperative limitation. The mean range of motion at the time of final follow-up was significantly improved compared with the preoperative findings in terms of forearm pronation, supination, and elbow flexion (*p* < 0.05) (Tables 3 and 4). However, there was no statistically significant improvement in the other ranges of motion.

### Complications

Some complications were observed during the treatment period. Fracture of the regenerate ulna after removing the external fixation was observed in case 1. The fracture was repaired by a cast for 4 weeks, and solid osseous union was ultimately achieved. Ulnar shorting recurred in case 6, 8, 11, 15 and 18 at the final follow-up. Pin tract infection was seen in case 3 and cured by local pin site care and oral antibiotics.

## Discussion

MHE was first reported by Boyer in 1814^18^. In the 1920s, some authors assured its hereditary by analysis of the family history; subsequently, some reports found that sarcomatous transformation occurs at a mean age of 20–40 years in multiple hereditary exostoses^19–21^. The main change of MHE is lesion appearance and function. Prichett reported that the forearm deformity caused by shortening of the ulnar relative to the radius in patients with multiple hereditary exostosis is related to three factors^13^_._ First, the distal ulnar physis has only a one-quarter cross-sectional area compared with the distal radius, meaning the distal ulnar physis growth can be more severely affected by the disease in the wrist. Second, the distal ulna is more commonly affected in the condition than the distal radius; thus, the distal ulnar physis is more susceptible. Third, there is more longitudinal growth capacity at the distal ulnar physis than at the distal radial physis, with the contribution of the distal ulnar physis to total ulnar length versus the distal radius physis to total radius length being 85 and 75% respectively.

Deformity of the forearm is common in patients with hereditary multiple exostoses. The most affected mobility preoperatively was prona-supination, especially for the Masada type 2 deformity. Clement studied forearm function in 106 patients who were fifteen years of age or older and showed that both radial head dislocation and proportional ulnar length were independent risk factors associated with forearm rotation on multivariate regression analysis^7^_._ In particular, a proportional ulnar length of < 13% in males and < 12% in females was associated with a forearm range of motion of < 100°, which limits activities of daily living. In addition, radial head dislocation was an additive factor associated with a further diminished range of motion, independently reducing forearm rotation by 36°. In our research, we found that preoperative forearm pronation was more impaired than supination, and elbow extension was not limited preoperatively or postoperatively.

Although several techniques for the surgical treatment of forearm deformities have been described, the operative treatment for the indication, time and standard procedure of Masada type 2 deformity remains unclear. Mader recommended the indications to be shortening of the radius or ulna by more than 2 cm, symptomatic loss of wrist movement, forearm and elbow movement and carpal slip of over 50% and RAA of over 40°^22^_._ Akita reviewed 23 patients after a mean follow-up 19 years and concluded that the reasonable indications for forearm surgery were to improve forearm rotation and to improve the appearance^23^_._ In our research, we suggested the indications for correction forearm deformity to be the dislocation of radial head combined with one of the following: ulna shorting by more than 1 cm, RAA of over 30°, carpal slip of over 60%, forearm rotation limited and painful exostosis or prominent osteochondromas causing neural or vascular compression. There are 2 opposing opinions about the time of surgery. Several authors have recommended early and aggressive surgical intervention because of their patients’ satisfaction of acquired forearm appearance and function, but the mean follow-up of their patients was too short to assess recurrences^1,12,24^_._ The second view represented by Donald et al. proposed a less aggressive treatment in the early and recommended simple excision of symptomatic osteochondromas through a long period of follow-up to skeletal maturity^25^. In our research, we suggest that aggressive corrective treatments, such as ulnar distraction osteogenesis and radial osteotomy, should be operated after 10 years because of the recurrence of ulnar shorting. However, once the radial head dislocates, surgical attempts at relocation should be exerted quickly because radial head dislocation is associated with reduced forearm rotation and functional impairment, which affect daily activity.

In 1989, Masada classified the forearm deformity caused by multiple heredity osteochondromas into three types and described a surgical treatment guideline for the forearm deformities: excision of osteochondromas, immediate ulnar lengthening, and corrective osteotomy of the radius for Type 1 deformity^12^_._ Excision of the radial head is necessary for Type 2A, gradual lengthening of the ulna using an external fixator for Type 2B, and excision of osteochondromas alone in Type 3 deformities. He confirmed that satisfactory results were achieved in 92% of cases using this technique. Shin compared the clinical outcomes of simple exostosis excision, ulnar lengthening and the Sauvé-Kapandji procedure in the treatment of deformities of the forearm in patients with multiple heredity osteochondromas^26^_._ They found that simple excision may improve the range of movement of the forearm but will not halt the progression of disease, particularly in younger patients. No discernible clinical or radiological improvement was noted with ulnar lengthening, and the Sauvé-Kapandji procedure combined with simple excision of osteochondromas could improve stability of the wrist, movement of the forearm and the radiological appearance. Kelly performed a retrospective review of 18 forearms affected by multiple heredity osteochondromas that underwent radial hemiepiphyseal stapling with a follow-up of over 2 years and acquired excellent results^27^_._ In addition, they concluded that hemiepiphyseal stapling of the radial side of the distal radius was a simple and effective method for correcting the radiographic deformity of the distal radius and may be an attractive option for the treatment of the distal radial deformity.

The treatment of the forearm deformity of MHE continues to be controversial, but there is consensus on the need for lengthening and normalization of the relationship of the radius and ulna^28^_._ For Masada type 2 deformity, the main keystone of treatment is reduction of the radial head and correction of the ulnar shorting. Demir et al. reported a successful outcome of the treatment of complete radial head dislocation with gradual ulnar lengthening using an external fixator^29^_._ They stated that the radial head was gradually transferred to the “reduction localization” along with ulnar lengthening, and a fibrous bed around the radial head increased the stability, demonstrated in their study using magnetic resonance imaging. However, the ulnar lengthening process was terminated when the radial head came to the same level as the coronoid process on the lateral view of the X-ray, and the ulnar length could not be effectively restored. In our study, after gradual ulnar distraction at a rate of 1 mm/day, the radial head was located at the same level as that of the coronoid process on a lateral radiogram, and we adjusted the K-wire to continue to lengthen the ulnar by 5 mm plus variance. We performed stage-by-stage ulnar lengthening by an Ilizarov ring fixator to reduce the radial head and restore the ulnar length, thus ensuring successful clinical and radiological results.

Additionally, most of the gradual ulnar lengthening procedures were performed using unilateral rail fixators, and there were only a few reports regarding the use of the Ilizarov technique for these procedures^30–32^. In the process of ulnar lengthening, the unilateral external fixator reduces the dislocated radial head through the interosseous membrane to drag the radius distally, and the distal radioulnar joint cannot be normalized, which theoretically diminishes ulnar support of the carpus and increases ulnar sided pressure on the radial epiphysis. However, the Ilizarov technique can first directly fix and drag the distal radius to reduce the dislocated radial head and then adjust the K-wire (remove the distal radius fixation and fix the proximal radioulnar joint) to second, continue the ulnar lengthening to normalize the distal radioulnar joint. In addition, the Ilizarov circular frame is more stable and can correct multi-planar deformity, and we recommended it for the surgical treatment of Masada type 2 deformity.

In this study, we achieved satisfactory clinical and radiological results with the modified ulnar lengthening for correction of Masada type 2 forearm deformity in hereditary multiple exostosis with the Ilizarov technique. In the latest follow-up, no patient reported radial head dislocation. Only 5 patients showed recurrent ulnar shortening and we continued to closely monitor the ulnar shorting without needing surgical intervention. There was also one case with a pin-track infection that was well controlled by pin care and oral antibiotics. Fracture occurred in one case after frame removal but healed after 4 weeks of casting.

## Conclusion

Modified ulnar lengthening by an Ilizarov external fixation with tumour excision for the treatment of Masada type 2 forearm deformities in Hereditary Multiple Exostoses has proven to be an effective and reliable technique in the early stage. This technique can relocate the radial head and normalize the distal radioulnar joint, improving the upper extremity function and appearance. Thus, we recommend this technique for the correction of Masada type 2 forearm deformity in hereditary multiple exostosis.

## Materials and methods

### General information

We retrospectively reviewed the patient database at our hospital between 2014 and 2021. This retrospective study was conducted in accordance with the Declaration of Helsinki and received approval from the Ethics Committee of Hunan Provincial People’s Hospital. Written informed consent was acquired from all patient parents and/or legal guardians. We retrospectively reviewed 20 patients with hereditary multiple exostoses who had radial head dislocation managed with modified ulnar lengthening according to the reduction of the radial head and the correction of abnormalities of the distal radioulnar joint. There were 13 girls and 7 boys, ranging in age from 3.5 to 15 years (mean: 9 years) at the time of operation. The deformations were present in 8 left forearms and 12 right forearms, and restriction of pronation/supination of the forearm before surgery existed in all cases. Prior to operation, anteroposterior and lateral X-ray of both forearms were obtained for all patients. Ulnar shortening (US), the radial articular angle (RAA) and carpal slip (CS) were measured and recorded as described by Fogel^1^. According to the forearm morphological evaluation system made by Masada et al., 9 forearms were Masada type 2a deformity, and 11 forearms were Masada type 2b deformity^12^. The follow-up period ranged from 24 to 60 months (mean: 36 months) after removing the external fixation. All patient parents and/or legal guardians involved in this study gave informed written consent to participate.

### Operative technique

**Figure 1:**
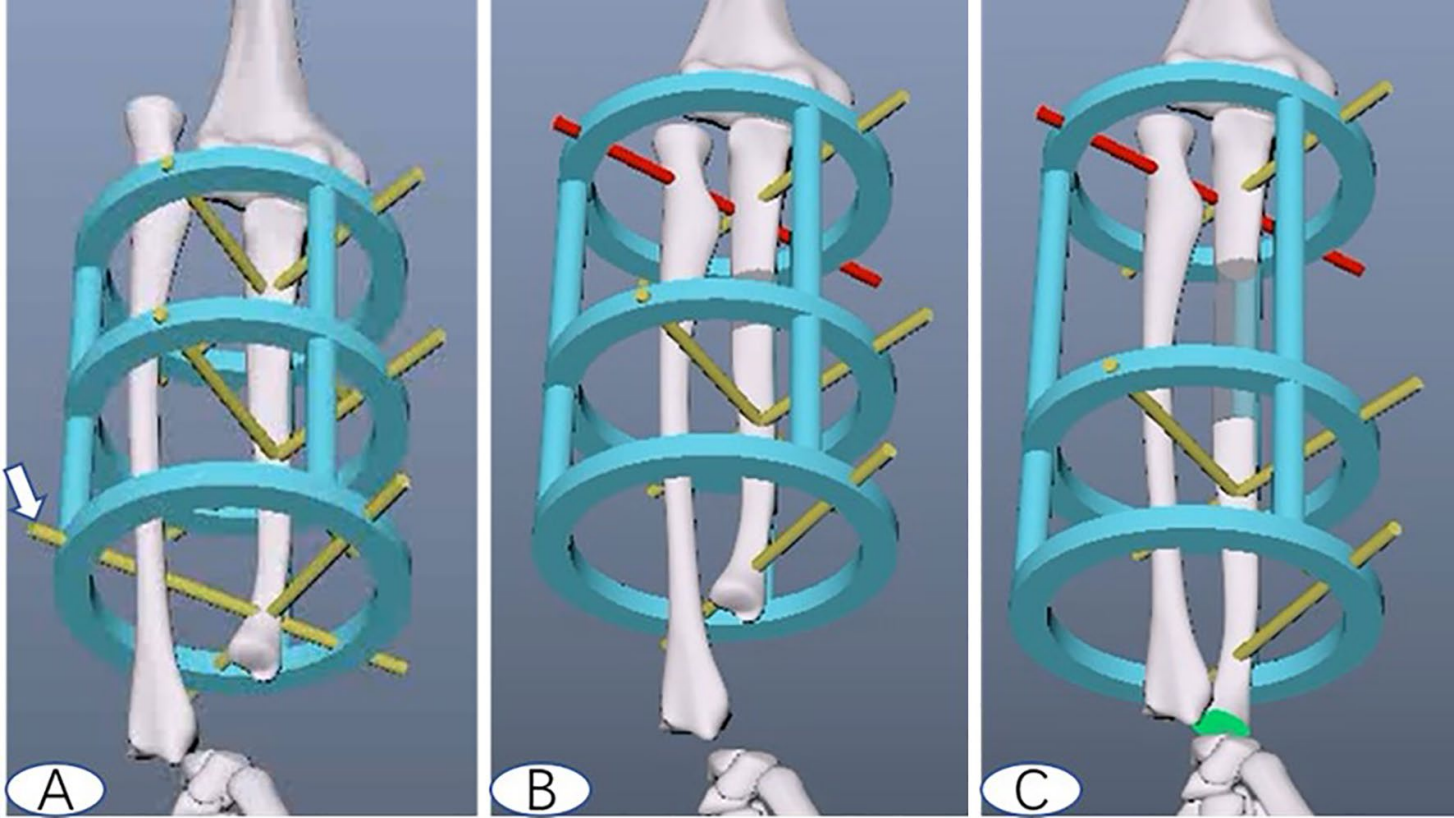
The diagrams show the process of modified ulnar gradual lengthening. Noticed one K-wire (arrow) traversed the distal of ulnar and radius, the others just pined on the ulnar (**A**), ulnar osteotomy between the first and the second ring. When the radial head came to same level with the coronoid process, removed the distal radioulnar K-wire, one proximal ulnar K-wire fixation was instead by one proximal radioulnar K-wire immobilization (the red K-wire, **B**). Continued lengthen the ulnar to 5 mm plus variance (the green part, **C**).

The operative technique was as follows. First, we removed the prominent osteochondromas of the distal ulna and the proximal radius. Second, we positioned a classic Ilizarov external fixator on the forearm, which was made up of three circular rings and rods. The inner diameter of the ring was 4 cm more than the diameter of the forearm to allow swelling after the operation, and the length of the rod was 5 cm longer than the length of the forearm. The first ring was located in the proximal ulna, which was fixed by two crossed 2.0 mm diameter Kirschner wires; the second ring was applied over the same type K-wires for fixation of the osteotomized ulnar distal segment. The distance of the first and second ring was preserved enough to achieve ulnar osteotomy. The third ring was immobilized by two vertical 2.0 mm diameter K-wires, one that traversed the distal ulna and radius and the other only fixing the distal ulna (Fig. 1A). Then, we performed ulnar transverse one-third proximal diaphyseal subperiosteal osteotomy through multiple drill holes with preservation of the periosteum, which was performed between the first and second ring.

### Postoperative management

The anteroposterior and lateral view X-rays of the forearm were filmed for all children on the first day after operation for comparison with subsequent images. Gradual ulnar distraction and pulling down of the radius were initiated at a rate of 1 mm per day after a latent period of 7 days. The first stage of ulnar lengthening was temporarily terminated when the radial head came to the same level as the coronoid process on the lateral view of the X-ray, indicating radial head reduction. Then, the distal radioulnar K-wire fixation was removed; in the same session, one proximal ulnar K-wire fixation was instead by one proximal radioulnar K-wire immobilization (Fig. 1B). We continued the second stage of ulnar lengthening by 5 mm plus variance for paediatric patients, with the prevention of subsequent recurrence of ulnar shortening (Fig. 1C). Patients were encouraged to use their wrists, forearms and elbows during the process. External fixators were removed upon completion of the consolidation phase, and physical rehabilitation was conducted for an additional 3–6 weeks.

Complications, including nerve palsies, tightness of the flexor tendons, pin track sepsis, ulnar regenerate callus fracture and ulnar shorting recurrence, were recorded during the treatment.

### Efficiency assessment

The patients were evaluated as outpatients every 3 months after removing the external fixation for the first year; then, a 6-month outpatient visit was requested. Clinical functions were assessed according to the latest follow-up with range of motion of the wrist, forearm and elbow by the same physician who had not been involved in the surgical procedures. X-ray examinations were performed to compare the changes of ulnar shortening (US), radial articular angle (RAA), carpal slip (CS), and reduction of the radial head. We also evaluated the amount of ulnar lengthening, external fixation time (EFT), external fixation index (EFI), and distraction index (DI). EFI was obtained by dividing the total duration of external fixation by the length gained. DI was obtained by dividing the total duration of distraction by the length gained.

### Statistical analysis

Data were analysed using SPSS statistical analysis software programme version 15.0 (SPSS Inc., Chicago, IL, USA). Continuous variables were summarized as the means and the standard deviations (SD). Student’s t test was used to compare paired data. Statistical significance was considered at *p* ≤ 0.05.

### Case report

**Figure 2:**
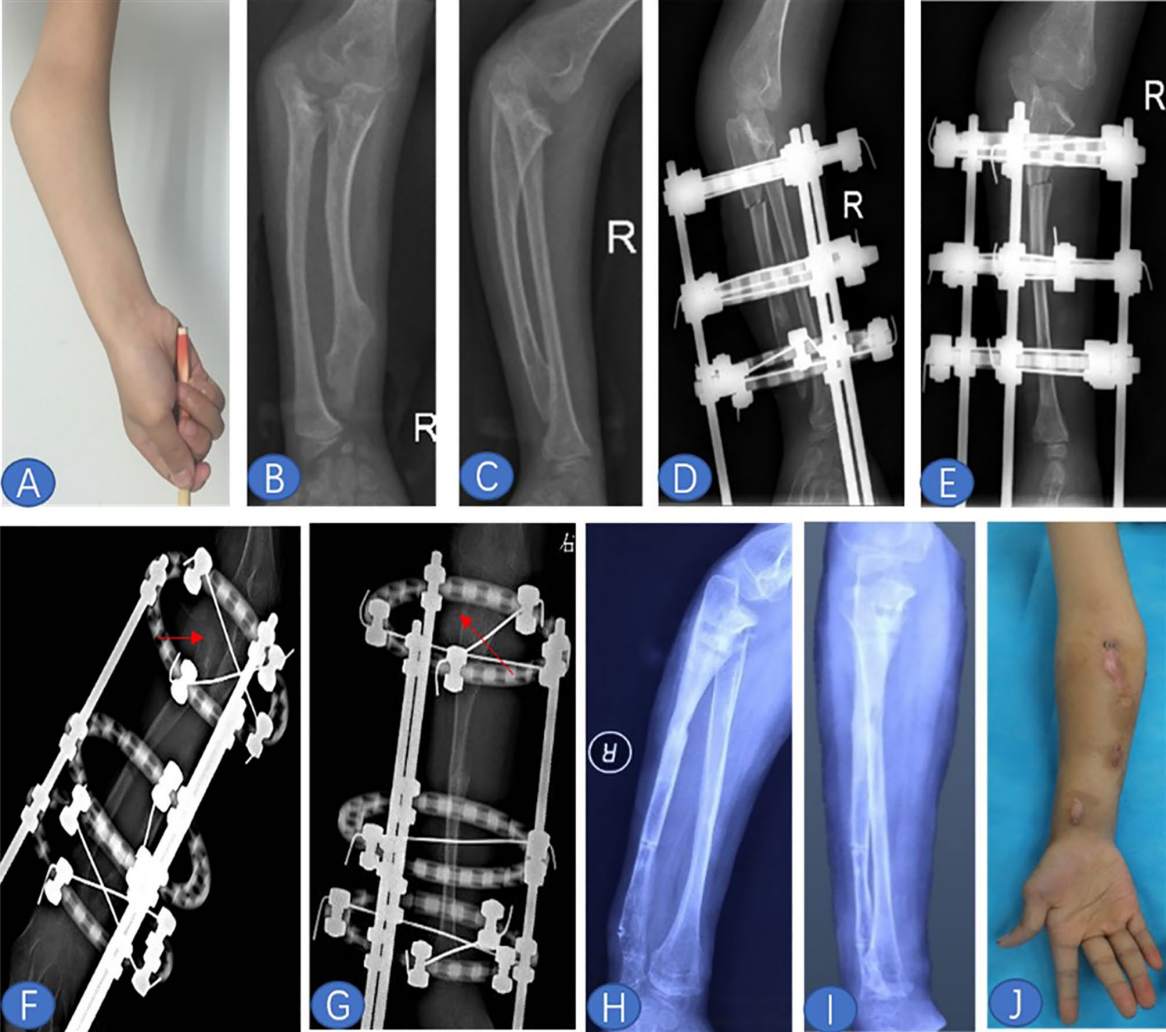
Functional and cosmetic improvement were achieved in all cases. Preoperative cosmetic (**A**) and radiological (**B**, **C**), the anteroposterior (**D**) and lateral (**E**) view X-ray of the forearm on the first day after operation, the radial head distracted at the same level with the coronoid process (red arrow in **F**, **G**) adjust the K-wire (remove the distal radius fixation and fix the proximal radioulnar joint), and postoperative radiological (**H**, **J**) and cosmetic (**J**) views of case 5.

Case report (case 5) is shown in Fig. 2.

## Data Availability

The datasets used and/or analysed during the current study available from the corresponding author on reasonable request.
